# Determinants of In-Stent Restenosis in ST-Elevation Myocardial Infarction: Insights from a Single-Center Retrospective Analysis

**DOI:** 10.3390/medicina62040785

**Published:** 2026-04-19

**Authors:** Alice Elena Munteanu, Alexandru Andrei Badea, Silviu Marcel Stanciu, Alexandru Mihai Popescu, Florentina Cristina Pleșa, Ciprian Constantin

**Affiliations:** 1 Department of Medical-Surgical and Prophylactical Disciplines, Faculty of Medicine, “Titu Maiorescu” University, 031593 Bucharest, Romania; 2 “Carol Davila” Emergency Hospital, 88 M. Vulcanescu Street, 010825 Bucharest, Romania; 3 Doctoral School, Faculty of Medicine, “Carol Davila” University of Medicine and Pharmacy, 050474 Bucharest, Romania; 4 Department No. 5—Internal Medicine, “Carol Davila” University of Medicine and Pharmacy, 050474 Bucharest, Romania; 5 Department No. 6—Clinical Neurosciences, “Carol Davila” University of Medicine and Pharmacy, 050474 Bucharest, Romania; 6 Research Metabolism Center, Faculty of Medicine, Titu Maiorescu University, 76 Al I Cuza Blvd, 011053 Bucharest, Romania

**Keywords:** restenosis, stenting, PCI, STEMI, inflammation, diabetes, thrombolytic

## Abstract

*Background and Objectives*: Percutaneous coronary intervention (PCI) has markedly improved outcomes in coronary artery disease through the implantation of bare-metal stents (BMS) or drug-eluting stents (DES). However, in-stent restenosis (ISR) remains a significant complication, often necessitating repeat interventions. This study aimed to identify risk factors associated with ISR in patients with ST-elevation myocardial infarction (STEMI) who underwent PCI. *Materials and Methods*: We conducted a retrospective, non-randomized observational study of 107 STEMI patients treated with PCI between January 2016 and December 2019 who subsequently underwent clinically indicated (predominantly symptom-driven) follow-up coronary angiography within 12 months. ISR was defined as ≥50% luminal narrowing at follow-up angiography. Time-to-event analysis was performed using Cox regression models, incorporating clinical, biochemical, and angiographic variables. *Results*: In this selected cohort of patients undergoing follow-up angiography, ISR of any degree was identified in 87% of patients, and 52% had restenosis >70%. Advanced age, prior cardiovascular events, diabetes mellitus, chronic kidney disease, and history of stroke significantly increased the hazard of ISR. Smoking, dyslipidemia, and hypertension were prevalent in patients with severe ISR. Women presented with more severe clinical profiles (higher Killip class and troponin levels). DES showed slightly better TIMI flow than BMS, but stent type, dimensions, and number did not significantly impact restenosis risk. Thrombolytic therapy was associated with a significantly reduced ISR hazard. Mortality was 6% in patients with severe ISR. The highest restenosis incidence occurred in the LAD and RCA territories. *Conclusions*: ISR is a multifactorial process influenced by demographic, clinical, and procedural factors. Despite technological advances, ISR remains a prevalent issue, particularly in high-risk groups undergoing clinically indicated follow-up angiography. Secondary prevention strategies, optimized stent deployment, and targeted therapies addressing inflammation and vascular remodeling are essential to improving long-term PCI outcomes.

## 1. Introduction

Percutaneous coronary intervention (PCI) has substantially improved the management of coronary artery disease, but symptom recurrence after apparently successful revascularization remains common. Persistent or recurrent angina after PCI affects a considerable proportion of patients during short- to medium-term follow-up and reflects a heterogeneous set of mechanisms, including incomplete revascularization, progression of native atherosclerotic disease, functional coronary abnormalities, stent thrombosis, and in-stent restenosis (ISR) [1,2].

Among the structural causes of post-PCI ischemia, ISR remains a clinically relevant form of stent failure. In the setting of coronary stenting, restenosis is primarily driven by neointimal hyperplasia in earlier phases and by neoatherosclerosis over time [2]. Although the introduction of drug-eluting stents (DES) has markedly reduced restenosis compared with bare-metal stents (BMS), ISR continues to account for a meaningful proportion of contemporary PCI activity. Large-scale data from the United States indicate that approximately 10% of PCI procedures are still performed for ISR, while pooled trial data show that restenosis-related target lesion revascularization remains detectable even with newer-generation DES during long-term follow-up [3,4,5,6].

The risk of ISR is not uniform and appears to depend on a combination of patient-related and procedure-related factors. Available evidence has linked ISR to diabetes mellitus, chronic kidney disease (CKD), smoking, hypertension, and inflammatory burden, as well as to angiographic or procedural variables such as stent type, low implantation pressure, and multi-stenting [4]. These associations are biologically plausible because endothelial dysfunction, vascular inflammation, impaired healing, and maladaptive neointimal response all contribute to restenosis after PCI [1,2,4]. From a clinical standpoint, identifying these determinants is important because they may help distinguish patients in whom recurrent ischemia is more likely to reflect stent-related failure rather than other causes of post-PCI angina [1,2].

ISR is also clinically important because it is not a benign entity. Compared with PCI for de novo coronary lesions, PCI for ISR is associated with a higher incidence of adverse events, including myocardial infarction, target vessel revascularization, and stent thrombosis [7]. Thus, beyond describing its occurrence, it is essential to define the risk profile of patients who develop ISR after PCI. In this context, the present study aimed to identify the clinical, biochemical, and angiographic factors associated with ISR in patients with ST-elevation myocardial infarction treated with PCI.

## 2. Materials and Methods

This retrospective, non-randomized observational study included 107 patients with STEMI who underwent PCI between January 2016 and December 2019 and who subsequently underwent clinically indicated follow-up coronary angiography within 12 months. Thus, the analyzed cohort represents a selected subgroup of STEMI patients referred for repeat angiographic evaluation, rather than all STEMI patients treated at our center during the study period (Table 1). Clinically indicated (predominantly symptom-driven) follow-up evaluation was performed to identify cases that subsequently developed ISR. Indications of follow-up angiography are described in Table 2. The primary objective was to identify risk factors for ISR. ISR was classified as non-significant (<50%), significant (≥50%), and severe (≥70%), based on the degree of arterial lumen obstruction. A time-to-event analysis was employed, with the event of interest being the presence or absence of stenosis. ISR was assessed through follow-up coronary angiography performed within 12 months after PCI.

Age was recorded as a continuous variable. For subgroup analysis, patients were additionally stratified according to an age cutoff of 67 years, which corresponded to the median value in the study cohort.

CKD was defined according to KDIGO criteria as abnormalities of kidney structure or function present for >3 months, with implications for health. In this cohort, because patients with severe renal dysfunction (eGFR < 30 mL/min/1.73 m^2^) were excluded, CKD in the analyzed population referred to previously documented non-severe CKD, i.e., eGFR 30–59 mL/min/1.73 m^2^ and/or other documented markers of kidney damage, when available.

Patients with severe chronic kidney disease (eGFR < 30 mL/min/1.73 m^2^) were excluded because the study design involved invasive coronary angiography, and repeated exposure to iodinated contrast media could substantially increase the risk of further renal function deterioration in this subgroup. This criterion was applied primarily for patient safety and to reduce the potential confounding effect of advanced renal dysfunction on clinical outcomes.

Left ventricular hypertrophy (LVH) was defined echocardiographically by increased left ventricular mass indexed to body surface area (LVMI), according to ASE/EACVI recommendations. Using linear measurements, LVH was defined as LVMI > 115 g/m^2^ in men and >95 g/m^2^ in women. If graded by severity, mild LVH corresponded to LVMI 116–131 g/m^2^ in men and 96–108 g/m^2^ in women, and moderate LVH to 132–148 g/m^2^ in men and 109–121 g/m^2^ in women.

Significant carotid stenosis was defined as ≥50% luminal narrowing of the internal carotid artery on carotid duplex ultrasonography or prior vascular imaging documentation; lesions below this threshold were classified as non-significant carotid plaque/stenosis. If a different duplex threshold was used by the vascular laboratory, that threshold should be stated explicitly.

Multivessel disease was defined as angiographically significant coronary artery disease involving ≥2 major epicardial vessels, generally corresponding to ≥70% diameter stenosis in non-left main vessels and/or ≥50% stenosis in the left main coronary artery.

Electrical complications were defined as clinically relevant rhythm and conduction disturbances occurring during STEMI, including atrial fibrillation, ventricular tachycardia, ventricular fibrillation, frequent ventricular extrasystoles/R-on-T phenomenon, sinus bradycardia, and atrioventricular conduction disorders (second-degree atrioventricular block type I or II and complete atrioventricular block).

Mechanical complications of STEMI were defined as post-infarction ventricular septal rupture, papillary muscle rupture with acute severe mitral regurgitation, and free-wall rupture or contained rupture with pericardial tamponade.

Cardiac troponin I was measured as high-sensitivity troponin I (hs-cTnI) using the PATHFAST assay platform, according to the manufacturer’s instructions and the hospital laboratory protocol.

Clopidogrel was mainly used when the more potent P2Y12 inhibitor (ticagrelor) is contraindicated, not tolerated, unavailable, or when bleeding risk is a major concern. Clopidogrel is also the default P2Y12 inhibitor when concomitant oral anticoagulation is required, Prasugrel was not available at our center during the study period.

Thrombolytic therapy was defined as administration of fibrinolytic treatment during the index STEMI event, prior to coronary angiography/PCI, typically in patients managed with a pharmacoinvasive strategy when timely primary PCI was not feasible. In our cohort, fibrinolysis was performed with alteplase using the accelerated STEMI regimen: a 15 mg intravenous bolus, followed by 0.75 mg/kg infused over 30 min (maximum 50 mg), then 0.5 mg/kg infused over the next 60 min (maximum 35 mg), up to a total maximum dose of 100 mg. This variable was recorded as a binary variable (yes/no) and included in the regression analysis.

This “pharmacoinvasive” approach implies that coronary angiography and subsequent PCI were performed as part of a structured protocol following fibrinolysis, which can include both immediate rescue PCI for failed reperfusion and routine early PCI (within 2–24 h) for successful lysis.

For the ISR model, the event was defined as angiographically confirmed ISR, and time was measured from stent implantation to restenosis detection. All demographic, clinical, laboratory, and angiographic variables were first evaluated using univariate Cox models. Variables with *p* < 0.05 in univariate analysis were incorporated into an initial multivariate model, followed by backward stepwise selection. Statistical analyses were performed in R version 4.0.2 (R Foundation for Statistical Computing, Vienna, Austria).

Based on information obtained from medical records at the time of control angiography, patients with ISR were divided into three categories according to their presentation: those with acute coronary syndrome (ACS) at onset; those with exertional angina, documented by exercise ECG or stress echocardiography; and those with newly developed arrhythmias, documented by 24 h Holter monitoring.

## 3. Results

The study included 107 patients with STEMI who underwent emergency coronary angiography within 24 h of symptom onset and primary PCI between January 2016 and December 2019, and who subsequently underwent clinically indicated follow-up coronary angiography (Table 3).

At presentation, they were between 36 and 78 years old. The mean time from index PCI to ISR detection at follow-up angiography was 204 days.

From the patient’s medical history, we learned that 29 (27%) people in the study group had a stent implanted before presenting with STEMI. The data obtained showed that 50 (47%) of the patients presenting with STEMI, although they had exertional angina, were not receiving specific treatment, increasing their risk of developing MI faster, due to the probable presence of an already formed atheromatous plaque.

**Figure 1:**
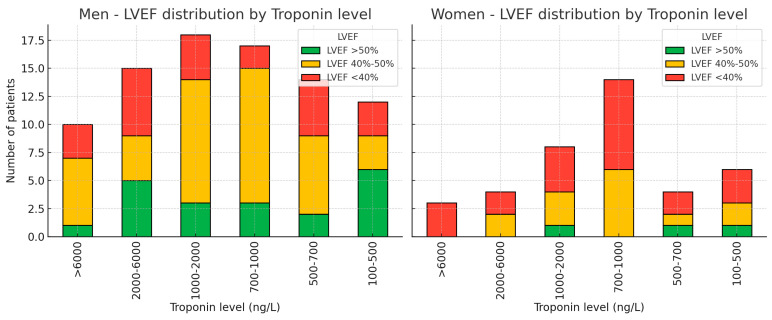
LVEF and troponin distribution. Note: LVEF = Left ventricle ejection fraction.

**Figure 2:**
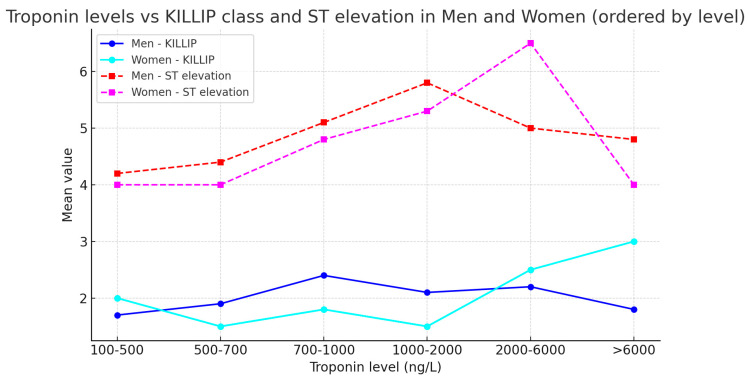
Killip class and ST elevation distribution.

Troponin had an average value between 1000 and 2000 ng/L in men, compared to women, with between 700–1000 ng/L, with correlations being made between this and LVEF (Figure 1). The distribution of LVEF associated with troponin demonstrates that LVEF in men is predominantly 40–50%, corresponding to a troponin level of 1000–2000 ng/L (Figure 2), respectively around 1000 ng/L (700–1000 ng/L) in women; thus, the higher the troponin level, the lower the LVEF. The maximum troponin value (500–1500) was at an interval between 4 and 7 h from the onset of pain in 27 (25%) patients.

A parameter used to assess the status of heart failure (HF) was the Killip class, upon admission of patients with STEMI-type ACS, and also subsequently, during hospitalization evaluated clinically and paraclinically. At the same time, upon admission of patients, an association was made between the Killip class, the amplitude of ST-segment elevation, and the serum troponin level, observing that women tend to have more pronounced symptomatology at presentation, having Killip class more frequently III or IV (Figure 2). The average ST-segment elevation was approximately 5 mm in men, with a Killip class at presentation of II, thus with mild symptomatology and a troponin level of approximately 1000–2000 ng/L. Comparatively, women had more severe symptomatology at admission and a ST-segment elevation level up to 6 mm and a higher troponin level compared to men, of 2000–6000 ng/L.

Averages of the BMS and DES stent characteristics (length, diameter, TIMI flow) used in the study show that BMS have a diameter of approximately 3.134 mm, a length of 17.58 mm, and TIMI 2.69, good post-angioplasty flow. At the same time, the same parameters were evaluated for DESs and it was observed that they have a diameter of 3.270 mm, a length of 19.904 mm, and a better flow of 2.825.

From the studied group with STEMI, it was analyzed which patients presented ISR—initially from the entire group, then by age and sex. Thus, 94 (87%) of the patients included in the study were detected with ISR of varying degrees at the control angiography (Table 2): 27 (29%) patients with non-significant stenosis (<50%), 18 (19%) patients with restenosis between 50% and 70%, and 49 (52%) patients with restenosis over 70%. Among patients with ISR, 68 underwent repeat revascularization, representing 63.6% of the ISR cohort.

The results of the univariate Cox regression analysis for clinical, biochemical, and angiographic predictors of ISR are presented in Table 4.

In the multivariable Cox model for ISR, prior stroke was independently associated with a higher hazard of angiographically detected ISR, whereas thrombolytic therapy was independently associated with a lower hazard of ISR. In a separate multivariable Cox model for the composite endpoint of death, recurrent myocardial infarction, or stroke, higher weight category, peripheral artery disease (PAD), coronary stenosis ≥45%, sartan therapy, and NYHA class III–IV were independently associated with a higher hazard of major adverse cardiovascular events.

On univariate Cox analysis, age ≥67 years, prior stroke, and thrombolytic therapy were significantly associated with ISR, while CKD showed a borderline association. In the multivariable ISR model, prior stroke remained independently associated with a higher hazard of ISR, whereas thrombolytic therapy was independently associated with a lower hazard of ISR; age ≥67 years did not remain significant after adjustment. Clinically, ISR most frequently presented as acute coronary syndrome. 

Variables with *p* < 0.05 in univariate analysis (age ≥ 67 years, previous stroke, thrombolysis) were entered into the multivariable Cox model (see Table 5).

Angiotensin II receptor blocker (ARB) therapy was associated with a threefold higher restenosis hazard (*p* < 0.01).

Mortality due to ISR was 6% in patients with significant restenosis and 2% in those with moderate restenosis.

In this part of the study (Table 6), the existence of predictive factors for the occurrence of severe complications (death, occurrence of a new myocardial infarction, occurrence of a stroke) over a period of one year after the placement of a coronary stent post-myocardial infarction was investigated. The percentage of events, the restricted mean of event occurrence, and the median of event occurrence (when possible) were calculated, these parameters being determined on the global group, as well as on various strata.

A multivariable Cox proportional hazards model was constructed (Table 7), including all variables with statistically significant associations in the preliminary analyses, and a backward selection algorithm was used to obtain the final model presented in Table 6. In this multivariable model, independent predictors of the hazard of severe events were higher body weight (approximately 2.3-fold increase in hazard), presence of PAD (approximately 2.7-fold increase), recurrent/residual coronary stenosis ≥45% documented at follow-up coronary angiography (approximately 5.5-fold increase), sartan therapy (approximately 2.7-fold increase), and advanced NYHA class III–IV (approximately 5.45-fold increase).

## 4. Discussions

### 4.1. Neointimal Hyperplasia

ISR reflects an abnormal vascular healing response after stent implantation, driven primarily by neointimal hyperplasia in earlier phases and by neoatherosclerosis over time. Mechanical vascular injury, inflammation, endothelial dysfunction, and vascular smooth muscle cell proliferation all contribute to luminal renarrowing after PCI [8,9,10]. These mechanisms are clinically relevant because they may be influenced by patient comorbidity burden, lesion characteristics, and procedural factors, which is consistent with the associations observed in our cohort. In particular, the higher risk observed in patients with prior cerebrovascular disease and CKD may reflect a broader vascular and inflammatory substrate predisposing to restenotic remodeling.

### 4.2. Neoatherosclerosis

Neoatherosclerosis is a late mechanism of stent failure that develops within the neointima and may contribute to recurrent ischemia and adverse outcomes after PCI [9,10]. OCT is particularly useful for identifying neoatherosclerosis and related features such as lipid-rich neointima, calcification, or intrastent thrombus [11,12]. These concepts are relevant to the interpretation of ISR because they highlight that restenosis is mechanistically heterogeneous and not solely determined by angiographic severity. However, intracoronary imaging was not systematically available in the present study, which limited direct mechanistic characterization of the restenotic lesions.

### 4.3. In-Stent Thrombosis

Stent thrombosis remains another important mechanism of stent failure and may present as ACS, particularly when associated with underexpansion, delayed healing, or persistent thrombotic substrate [13,14,15,16,17,18]. Although thrombosis was not a primary endpoint of the present study, its clinical overlap with ISR is relevant because recurrent ischemic presentation after PCI may reflect mixed mechanisms rather than isolated neointimal proliferation alone.

### 4.4. ISR Presentation

ISR usually occurs 3–12 months after stent implantation and remains a major cause of target lesion failure, contributing to about 20% of target lesion revascularization at 10 years [19]. Target lesion failure includes cardiovascular death, clinically driven target lesion revascularization, and target vessel myocardial infarction; stent/scaffold thrombosis is another important related endpoint [20].

ISR may present either as an acute coronary syndrome (ACS) or as a non-ACS manifestation, with the clinical pattern appearing to depend more on patient-related than device-related factors. ACS is the most frequent presentation, whereas the remaining cases usually present with recurrent stable angina or silent ischemia [3,19,21]. In patients treated with DES, ISR has been associated with a higher frequency of unstable angina than de novo stenosis, although myocardial infarction may be less common at presentation [22].

Acute presentation may reflect superimposed thrombus, marked neointimal proliferation, or both. Thrombotic findings are reported more often in ACS-related ISR than in non-ACS ISR, suggesting that flow-limiting diffuse restenosis may promote secondary thrombus formation [3]. Although DES and BMS differ in restenosis frequency and angiographic pattern, the clinical mode of ISR presentation is not clearly distinguishable between stent types [3,21].

Clinical presentation has prognostic relevance. ISR presenting as ACS is associated with higher rates of major adverse cardiovascular events and increased 1-year mortality compared with non-ACS ISR and with de novo lesions, independent of baseline comorbidity burden [3,21,23].

Coronary angiography remains the reference standard for diagnosis, while computed tomography coronary angiography may offer a useful noninvasive alternative in selected cases [24,25]. Intravascular imaging, particularly OCT and IVUS, has improved mechanistic characterization of ISR by identifying factors such as stent underexpansion, neointimal hyperplasia, peri-stent calcification, and multiple stent layers, all of which may adversely affect repeat PCI results [26,27,28]. However, evidence remains insufficient to establish a clear outcome benefit of routine intravascular imaging guidance in ISR [29].

### 4.5. ISR Risk Factors

In this study, we monitored patients with STEMI treated with PCI per primam and implantation of BMS or DESs, who later developed ISR, which was detected during coronary angiography as a stenosis of over 50% at the site of the implanted stent.

In our cohort, ISR most often presented as ACS, consistent with previous reports [21]. Most patients with moderate or severe ISR presented with ACS (41%), followed by stable angina (32%). ISR was most frequent in patients aged 60–70 years, whereas sex was not significantly associated with restenosis risk, in line with previous reports [14,30].

Traditional cardiovascular risk factors, including hypertension, smoking, obesity, and diabetes, were common in the cohort, but none showed an independent association with ISR in this analysis, despite their recognized relevance in other studies [31,32,33,34,35]

Among the analyzed comorbidities, prior stroke emerged as one of the most relevant findings, being associated with a significantly higher event rate and an approximately twofold increase in restenosis risk. CKD was associated with a higher burden of ISR and worse prognosis, with approximately twice as many major cardiovascular events and a threefold higher risk of complications, although its association with ISR was only marginally non-significant [36,37].

Prior thrombolysis during the index STEMI was associated with a lower risk of ISR and fatal/non-fatal events, suggesting a possible protective effect in patients treated with a pharmacoinvasive strategy. One possible explanation is that fibrinolysis, as part of a pharmacoinvasive strategy, may have contributed to earlier reperfusion and lower thrombotic burden before PCI, thereby reducing vascular injury and the subsequent restenotic response.

Advanced HF status also had prognostic value: NYHA III–IV and Killip III–IV were associated with a markedly higher risk of major cardiovascular events, although neither Killip class nor ejection fraction predicted ISR itself [4,38,39,40]. In addition, tachycardia at presentation was associated with a substantially higher risk of major adverse events [41].

Stent-related factors were also clinically relevant. Although DES were used more frequently than BMS, stent type was not significantly associated with ISR in this cohort. However, a prior history of stent implantation was associated with more ISR lesions and a 2.3-fold higher risk of severe events, supporting previous observations that a greater stent burden may increase restenosis risk [33,42]. Larger stent diameter was also associated with a higher risk of major cardiovascular events, while increasing stenosis severity was strongly associated with worse outcomes: each 1% increase in stenosis raised the hazard of severe events by 2%, and coronary stenosis >45% increased event risk more than fivefold [43,44].

Overall, our findings suggest that prior cerebrovascular disease, thrombolysis status, HF severity, and selected stent-related characteristics were more informative in predicting outcomes than conventional risk factors alone.

### 4.6. Practical Implications and Future Perspectives

ISR remains a clinically relevant complication after PCI, particularly in high-risk patients. Prior studies have shown higher restenosis and adverse event rates in patients with diabetes, CKD, prior stroke, small vessels, long lesions, and multiple previous stents. In our cohort, prior stroke, thrombolysis status, heart failure severity, and selected stent-related characteristics were the most informative predictors of outcomes. These findings support careful risk stratification, optimization of secondary prevention, and close clinical follow-up, while observational signals such as differences between ACE inhibitors and ARBs should be interpreted cautiously and should not alter guideline-directed therapy in the absence of randomized evidence.

Intravascular imaging with OCT or IVUS remains important for understanding the mechanism of ISR and stent failure, including stent underexpansion, neoatherosclerosis, or diffuse neointimal proliferation, and may improve lesion characterization and procedural optimization [45,46]. Although optimal ISR treatment depends on identifying the underlying mechanism, these considerations should be interpreted in the context of the present study, which did not include systematic intracoronary imaging. Artificial intelligence may further refine cardiovascular imaging by enabling more standardized calcium scoring and automated OCT-based assessment of stent coverage and vascular healing, although these applications still require external validation and appropriate clinical integration [47,48,49].

### 4.7. Limitations

Despite the insights provided by this study on the risk factors associated with ISR several limitations should be acknowledged. This study was a retrospective, non-randomized observational analysis with a relatively small sample size (107 patients). A larger, multi-center study with a greater number of participants could provide more generalizable results and improve statistical power.

The follow-up period was limited to 12 months, which may not be sufficient to fully capture the long-term incidence and progression of ISR. ISR can develop several years after stent implantation, necessitating extended follow-up periods for a more comprehensive assessment.

The study was conducted at a single center, which may limit the external validity and generalizability of the findings. Different patient populations, procedural techniques, and healthcare settings might influence ISR rates and associated risk factors.

The non-randomized nature of the study may introduce confounding variables that influence ISR occurrence. Although multivariate analysis was performed, unknown confounders may still be present.

The study included both BMS and DES of various generations, which may have different restenosis rates and mechanisms. Furthermore, inter-operator variability in PCI techniques might influence outcomes.

An additional limitation is the relatively high proportion of BMS in the study cohort (41.1%). Because BMS are associated with a higher restenosis risk than contemporary DES, and because DES are the current standard of care, this limits the direct applicability of our findings to modern PCI practice. Although stent type was not significantly associated with ISR in our analysis, this absence of association may reflect limited statistical power, the heterogeneous inclusion of BMS and DES of different generations, and the selection bias inherent to the clinically indicated follow-up angiography design.

While certain clinical and biochemical factors were analyzed, inflammatory and genetic markers that may influence ISR, such as C-reactive protein (CRP), IL-6, and genetic polymorphisms, were not systematically assessed.

Relevant angiographic and procedural determinants of ISR were not systematically assessed. Specifically, detailed data on lesion complexity, including calcified lesions, bifurcation lesions, and chronic total occlusions, as well as on lesion preparation before stent implantation, were not consistently available. Furthermore, no intracoronary imaging or coronary physiology data were available. Consequently, the mechanism of restenosis and the functional significance of the identified lesions could not be fully characterized. The incorporation of intravascular imaging modalities such as OCT or IVUS could have offered a more detailed understanding of restenosis mechanisms.

## 5. Conclusions

In this selected cohort of STEMI patients undergoing clinically indicated follow-up angiography after PCI, ISR remained frequent and appeared to be influenced mainly by comorbidity burden rather than by stent type or dimensions. Prior stroke independently predicted ISR, whereas thrombolytic therapy was associated with a lower ISR risk. Major adverse cardiovascular events were associated with higher weight, peripheral artery disease, coronary stenosis ≥45% at follow-up angiography, ARB therapy, and NYHA class III–IV. These findings support careful risk stratification, optimized secondary prevention, and closer follow-up in high-risk patients. Given the retrospective single-center design and symptom-driven follow-up, larger prospective studies are needed to confirm these associations.

## Figures and Tables

**Table 1:** Inclusion and exclusion criteria in the study.

Inclusion Criteria	Exclusion Criteria
Coronary ischemia—related symptoms in patients with a history of STEMI	Patients with suboptimal post-procedural angiographic results.
History of STEMI treated with percutaneous coronary intervention and stent implantation at the culprit lesion site.	Patients with chronic kidney disease and eGFR < 30 mL/min/1.73 m^2^.
Patients aged over 18 years, located in Romania.	Patients with documented thrombophilias (Factor V Leiden, antiphospholipid syndrome).
Availability of complete clinical, laboratory, and angiographic data for analysis	Patients with major cardiac events within the first month after the procedure.
	Patients with acute in-stent thrombosis.
	Patients with major procedural complications or technically unsuccessful stent implantation at the index PCI (as judged by the interventional cardiologist).
	Patients with stents implanted in arterial or venous grafts.

**Table 2:** Indications for Follow-up Coronary Angiography.

Indications for Follow-Up Coronary Angiography
Recurrent chest pain with typical or atypical angina-like features.
Angina equivalents (fatigability, exertional dyspnea, newly occurring rhythm disturbances possibly of ischemic origin, new wall motion abnormalities, etc.).
Dynamic changes on resting ECG observed during follow-up examinations.
Dynamic changes on resting echocardiography (new wall motion abnormalities of the left ventricle, reduction in left ventricular ejection fraction, restrictive motion of the posterior mitral leaflet, etc.).
Positive stress tests (ECG-based or echocardiographic).
Findings suggestive of silent ischemia on 24 h continuous ECG monitoring (ventricular extrasystoles with a tendency to cluster, horizontal or downsloping ST-segment depression, etc.).
Signs of congestive heart failure (new onset or worsening of pre-existing symptoms).
In the context of percutaneous myocardial revascularization of another pre-existing coronary lesion.
In certain cases, angiography was repeated as part of invasive evaluation during pre-operative protocols for cardiac or non-cardiac surgery.

**Table 3:** Distribution of STEMI patients based on anamnesic, clinical, and paraclinical parameters.

Category	Subcategory	Number (%)	Category	Subcategory	Number (%)
**Sex**	Male	86 (80.37%)	**Anterior STEMI**	LAD	71 (66.36%)
	Female	21 (19.63%)		LMCA	5 (4.67%)
**Age (years)**	38–48	9 (8.41%)		D1	3 (2.8%)
	49–59	35 (32.71%)		Cx	6 (5.61%)
	60–70	46 (42.99%)		RCA	3 (2.8%)
	71–78	17 (15.89%)		M1	2 (1.87%)
**Hypertension**		86 (80.37%)	**Inferior STEMI**	LAD	2 (1.87%)
**LVH**	None	22 (20.56%)		LMCA	2 (1.87%)
	Mild	73 (68.22%)		D1	-
	Moderate	12 (11.21%)		Cx	3 (2.8%)
**BMI**	18.5–24.9	15 (14.02%)		RCA	39 (36.45%)
	25–29.9	41 (38.32%)		M1	2 (1.87%)
	>30	48 (44.86%)	**Number of stents**	1	84 (77.57%)
**Smoking**		92 (85.98%)		2	22 (20.56%)
**Dyslipidemia**		77 (71.96%)		3	1 (0.93%)
**Diabetes**	Without Insulin treatment (oral treatment)	26 (24.3%)	**Stent type**	BMS	44 (41.12%)
	Insulin treatment	13 (12.15%)		DES	63 (58.88%)
**CKD**		18 (16.82%)	**Stent size**	Diameter < 2.5 mm	4 (3.74%)
**Stroke**		16 (14.95%)		Diameter > 2.5 mm	42 (39.25%)
**Carotid Plaque**	Non-Significant	70 (65.42%)		Length < 30 mm	44 (41.12%)
	Significant Stenosis	18 (16.82%)		Length > 30 mm	3 (2.8%)
**PAD**		31 (28.97%)	**Previous stent**		29 (27.1%)
**hs-cTnI**	>6000	12 (11.21%)	**Thrombolysis**		19 (17.76%)
	1000–6000	39 (36.45%)	**TIMI Flow**	0	3 (2.8%)
	500–1000	41 (38.31%)		1	4 (3.74%)
	100–500	15 (14.02%)		2	9 (8.41%)
**LVEF**	>50%	23 (21.5%)		3	91 (85.05%)
	40–50%	57 (53.27%)	**Restenosis**	<50%	27 (25.23%)
	35–39%	17 (15.89%)		50–70%	18 (16.82%)
	30–34%	6 (5.61%)		>70%	49 (45.79%)
	<29%	4 (3.74%)	**Clinical Presentation**	ACS	27 (25.23%)
**Killip Class**	I	22 (20.56%)		Stable Angina	21 (19.63%)
	II	64 (59.81%)		AF	9 (8.41%)
	III	8 (7.48%)	**Multivessel Disease**		80 (74.77%)
	IV	3 (2.8%)	**ACEi**		76 (71.03%)
**NYHA class**	I	5 (4.67%)	**ARB**		21 (19.63%)
	II	45 (42.06%)	**Statin**	Atorvastatin	87 (81.31%)
	III	8 (7.48%)		Rosuvastatin	20 (18.69%)
	IV	2 (1.87%)	**CAI**		6 (5.61%)
**STEMI Localization**	Anterior	54 (50.47%)	**DAPT**	ASA + Clopidogrel	45 (42.06%)
	Inferior	43 (40.19%)		ASA + Ticagrelor	48 (44.86%)
	Lateral	12 (11.21%)		Aspirin + Clopidogrel + Anticoagulant	14 (13.08%)
	RV	6 (5.61%)	**Complications**	Electrical	23 (21.5%)
				Vascular	16 (14.95%)
				Death	4 (3.74%)

Note: LVH—Left Ventricular Hypertrophy, BMI—Body Mass Index, CKD—Chronic Kidney Disease, PAD—Peripheral Artery Disease, hs-cTnI—high-sensitivity cardiac troponin I, LVEF—Left Ventricular Ejection Fraction, STEMI—ST-Elevation Myocardial Infarction, LAD—Left Anterior Descending artery, LMCA—Left Main Coronary Artery D1—First Diagonal artery, CAI—Cholesterol absorption inhibitor (e.g., Ezetimibe), Cx—Circumflex artery, RCA—Right Coronary Artery, M1—First Marginal artery, BMS—Bare Metal Stent, DES—Drug-Eluting Stent, TIMI—Thrombolysis In Myocardial Infarction, ACS—Acute Coronary Syndrome, AF—Atrial Fibrillation, NYHA—New York Heart Association, ACEi—Angiotensin-Converting Enzyme Inhibitors, ARB—Angiotensin II Receptor Blockers, DAPT—Dual Antiplatelet Therapy.

**Table 4:** Univariate Cox regression analysis for clinical, biochemical and angiographic predictors of ISR.

Parameter	Events (*n*/%)	Mean	Median [95% CI]	Cox Regression HR [95% CI]	*p*-Value
**Sex**					
- Male	52/86 (60.46%)	8.21	9.00 [8.00–10.00]	Reference	-
- Female	15/21 (71.42%)	8.06	8.00 [6.00–N/A]	1.01 [0.57–1.80]	0.9540
**Age**					
- Age (≥67)	25/30 (83.33%)	7.00	8.00 [5.00–11.00]	1.83 [1.11–3.01]	0.0170
**Hypertension**	57/86 (66.27%)	8.06	9.00 [8.00–10.00]	1.45 [0.73–2.85]	0.2780
**Diabetes**	29/38 (76.31%)	7.64	9.00 [6.00–10.00]	1.45 [0.89–2.36]	0.1260
**Insulin treatment**	9/13 (69.23%)	8.25	9.00 [6.00–N/A]	1.18 [0.58–2.38]	0.6450
**BMI over 25**	55/78 (70.51%)	6.5	6.5 [5.7–7.2]	1.27 [0.92–1.76]	0.1430
**Smoking**	51/86 (59.30%)	8.19	9.00 [8.00–10.00]	0.96 [0.54–1.69]	0.9000
**Dyslipidemia**	49/77 (63.63%)	6.73	6.00 [4.5–7.0]	1.003 [0.78–1.28]	0.9830
**HDL < 60 mg/dL**	58/87 (66.67%)		N/A	1.08 [0.80–1.45]	0.5850
**CKD**	18/23 (78.26%)	6.40	5.00 [4.00–11.00]	1.68 [0.97–2.89]	0.0594
**Stroke**	12/16 (75.00%)	6.15	5.00 [3.00–N/A]	2.21 [1.16–4.21]	0.0149
**Carotid Plaque**	58/88 (65.91%)		N/A	1.07 [0.70–1.64]	0.7360
**PAD**	22/31 (70.96%)		N/A	1.45 [0.87–2.43]	0.1520
**AF**	9/14 (64.28%)	6.48	6.00 [5.00–N/A]	2.01 [0.97–4.13]	0.0570
**Troponin Levels**	N/A		N/A	1.00 [1.00–1.00]	0.4170
**ST Segment Elevation**	N/A		N/A	1.09 [0.91–1.30]	0.3100
**Killip Class**					
- Killip 1 & 2	55/88 (62.50%)		N/A	Reference	-
- Killip 3 & 4	12/19 (63.15%)		N/A	1.32 [0.93–1.87]	0.1190
**LVEF**	N/A		N/A	1.23 [0.96–1.59]	0.0957
**Stent Type**					
- BMS	26/44 (59.09%)	8.83	9.00 [9.00–12.00]	Reference	-
- DES	41/63 (65.07%)	7.75	8.00 [7.00–10.00]	1.30 [0.79–2.13]	0.2980
**Stent Diameter**	N/A		N/A	1.04 [0.99–1.10]	0.1070
**Stent Length**	N/A		N/A	1.04 [0.99–1.10]	0.1070
**Number of Stents**	N/A		N/A	1.15 [0.65–2.03]	0.6120
**Previous Stent**	18/23 (78.26)	6.81	6.00 [4.00–10.00]	1.64 [0.95–2.83]	0.0702
**TIMI Flow**	N/A		N/A	1.10 [0.77–1.57]	0.5840
**Localisation of IM**					
- Anterior	34/54 (62.96%)		N/A	0.89 [0.55–1.44]	0.6570
- Inferior	26/43 (60.47%)		N/A	1.04 [0.86–1.25]	0.6520
- Lateral	8/12 (66.67%)		N/A	1.77 [0.84–3.74]	0.1320
- VD	4/6 (66.67%)		N/A	0.70 [0.25–1.95]	N/A
**Stented Artery**					
- LAD	32/50 (64.00%)		N/A	1.11 [0.81–1.50]	0.4960
- Left main	5/7 (71.43%)		N/A	1.23 [0.49–3.06]	0.6570
- CX	9/15 (60.00%)		N/A	1.20 [0.59–2.43]	0.6080
- Right Coronary	25/42 (59.52%)		N/A	1.15 [0.70–1.89]	0.5740
- Diagonal	2/6 (33.33%)		N/A	0.93 [0.33–2.57]	0.8940
- Marginal	4/4 (100.00%)		N/A	0.57 [0.14–2.36]	0.4460
**Thrombolysis**	6/19 (31.57)	9.50	N/A [7.00–N/A]	0.41 [0.17–0.95]	0.0396
**Antiplatelet Therapy**					
- Clopidogrel	37/54 (68.51%)	8.10	9.00 [7.00–12.00]	Reference	-
- Ticagrelor	30/53 (56.60%)	8.31	9.00 [8.00–11.00]	0.87 [0.53–1.41]	0.5800
**Statin Type**					
- Rosuvastatin	11/20 (55.00%)	8.90	9.00 [7.00–N/A]	Reference	-
- Atorvastatin	56/87 (64.39%)	8.05	9.00 [8.00–10.00]	1.20 [0.63–2.30]	0.5730
**ACE inhibitors**					
- No	19/31 (61.29%)	7.30	8.00 [5.00–N/A]	Reference	-
- Yes	48/76 (63.15%)	8.52	9.00 [8.00–11.00]	0.71 [0.41–1.21]	0.2170

Notes: CI—Confidence Interval, HR—Hazard Ratio, *p*-value—Probability value, BMI—Body Mass Index, HDL—High-Density Lipoprotein, CKD—Chronic Kidney Disease, PAD—Peripheral Artery Disease, AF—Atrial Fibrillation, LVEF—Left Ventricular Ejection Fraction, BMS—Bare-Metal Stent, DES—Drug-Eluting Stent, TIMI—Thrombolysis in Myocardial Infarction, IM—Myocardial Infarction, LAD—Left Anterior Descending Artery, CX—Circumflex Coronary Artery, ACE—Angiotensin-Converting Enzyme.

**Table 5:** Independent predictors of restenosis in the multivariable Cox proportional hazards model.

Parameter	Coefficient	*p* Value	HR [95% CI]
**Thrombolysis**	−1.11	0.0174	0.33 [0.13–0.82]
**Previous stroke**	0.86	0.0083	2.38 [1.25–4.5]

**Table 6:** Predictive factors for the occurrence of severe complications.

Parameter	Events (%)	Mean	Median [95% CI]	Cox Regression HR [95% CI]	*p*-Value
**Overall Lot**	26/107 (24.29%)	10.03	N/A [11.00–N/A]	N/A	N/A
**Age** **≥ 60 Years**	20/63 (31.74%)	9.37	11.00 [10.00–N/A]	2.70 [1.08–6.75]	0.0325
**Diabetes**	14/38 (36.84%)	9.36	11.00 [9.00–N/A]	2.20 [1.01–4.76]	0.0450
**Weight**	N/A	N/A	N/A	3.12 [1.51–6.46]	0.0021
**PAD**	15/31 (48.38%)	8.42	N/A [5.00–N/A]	3.86 [1.76–8.43]	0.0006
**CKD**	10/23 (43.47%)	8.37	9.00 [9.00–N/A]	2.90 [1.31–6.41]	0.0084
**Tachycardia**	24/81 (28.57%)	9.55	11.00 [11.00–N/A]	4.46 [1.05–18.88]	0.0423
**Killip Class**					
Killip 1 & 2	18/88 (20.45%)	10.39	N/A	Reference	-
Killip 3 & 4	8/19 (42.10%)	8.35	9.00 [5.00–N/A]	2.77 [1.20–6.41]	0.0168
**NYHA Class**					
NYHA 1 & 2	20/95 (21.05)	10.49	N/A [11.00–N/A]	Reference	-
NYHA 3% 4	6/12 (50.00%)	5.08	N/A [11.00–N/A]	5.14 [1.99–13.24]	0.0006
**Stent Diameter**					
<3 mm	4/29 (13.79%)	10.79	N/A	Reference	-
≥3 mm	22/77 (27.84%)	9.61	11.00 [10.00–N/A]	3.01 [1.02–8.89]	0.0456
**Degree of Stenosis**					
<45%	3/40 (7.50)	11.23	N/A [N/A–N/A]	Reference	-
>45%	23/67 (34.32)	9.80	N/A [10.00–N/A]	5.19 [1.55–17.35]	0.0073
**Previous Stent**	9/23 (39.13%)	9.00	11.00 [9.00–N/A]	2.29 [1.02–5.16]	0.0440
**ARB**					
No	16/83 (19.27)	10.57	N/A [N/A–N/A]	Reference	-
Yes	10/24 (41.66)	8.01	9.00 [5.00–N/A]	3.33 [1.49–7.44]	0.0032

Notes: CI—Confidence Interval, HR—Hazard Ratio, N/A—not available, PAD—Peripheral Artery Disease, CKD—Chronic Kidney Disease, NYHA—New York Heart Association, ARB—Angiotensin Receptor Blocker.

**Table 7:** Independent predictors of severe events in the multivariable Cox proportional hazards model.

Parameter	Coefficient	*p* Value	HR [95% CI]
**Weight**	0.83	0.0282	2.30 [1.09–4.87]
**PAD**	0.98	0.0198	2.68 [1.16–6.14]
**Stenosis ≥45%**	1.69	0.0066	5.45 [1.60–18.60]
**Sartan therapy**	1.01	0.0169	2.72 [1.20–6.40]
**NYHA class III–IV**	1.69	0.0012	5.45 [1.95–15.19]

Notes: PAD = peripheral arterial disease, NYHA = New York Heart Association functional class, CI = confidence interval, HR = hazard ratio.

## Data Availability

The data presented in this study are available on request from the corresponding author due to privacy reasons.

## References

[1] De Luca L., Rosano G.M., Spoletini I. (2022). Post-percutaneous coronary intervention angina: From physiopathological mechanisms to individualized treatment. Cardiol. J..

[2] Crea F., Merz C.N.B., Beltrame J.F., Berry C., Camici P.G., Kaski J.C., Ong P., Pepine C.J., Sechtem U., Shimokawa H. (2019). Mechanisms and diagnostic evaluation of persistent or recurrent angina following percutaneous coronary revascularization. Eur. Heart J..

[3] Moussa I.D., Mohananey D., Saucedo J., Stone G.W., Yeh R.W., Kennedy K.F., Waksman R., Teirstein P., Moses J.W., Simonton C. (2020). Trends and Outcomes of Restenosis After Coronary Stent Implantation in the United States. JACC.

[4] Alexandrescu D.-M., Mitu O., Costache I.I., Macovei L., Mitu I., Alexandrescu A., Georgescu C.A. (2021). Risk factors associated with intra-stent restenosis after percutaneous coronary intervention. Exp. Ther. Med..

[5] Madhavan M.V., Kirtane A.J., Redfors B., Généreux P., Ben-Yehuda O., Palmerini T., Benedetto U., Biondi-Zoccai G., Smits P.C., von Birgelen C. (2020). Stent-Related Adverse Events > 1 Year After Percutaneous Coronary Intervention. J. Am. Coll. Cardiol..

[6] Kastrati A., Cassese S. (2020). In-Stent Restenosis in the United States: Time to Enrich its Treatment Armamentarium. J. Am. Coll. Cardiol..

[7] Elbadawi A., Dang A.T., Mahana I., Elzeneini M., Alonso F., Banerjee S., Kumbhani D.J., Elgendy I.Y., Mintz G.S. (2023). Outcomes of Percutaneous Coronary Intervention for In-Stent Restenosis Versus De Novo Lesions: A Meta-Analysis. J. Am. Heart Assoc..

[8] Tucker B., Vaidya K., Cochran B.J., Patel S. (2021). Inflammation during Percutaneous Coronary Intervention—Prognostic Value, Mechanisms and Therapeutic Targets. Cells.

[9] Chen Z., Matsumura M., Mintz G.S., Noguchi M., Fujimura T., Usui E., Seike F., Hu X., Jin G., Li C. (2022). Prevalence and Impact of Neoatherosclerosis on Clinical Outcomes After Percutaneous Treatment of Second-Generation Drug-Eluting Stent Restenosis. Circ. Cardiovasc. Interv..

[10] Borovac J.A., D’Amario D., Niccoli G. (2017). Neoatherosclerosis and Late Thrombosis After Percutaneous Coronary Intervention: Translational Cardiology and Comparative Medicine from Bench to Bedside. Yale J. Biol. Med..

[11] Nagano Y., Otake H., Toba T., Kuroda K., Shinkura Y., Tahara N., Tsukiyama Y., Yanaka K., Yamamoto H., Nagasawa A. (2019). Impaired Cholesterol-Uptake Capacity of HDL Might Promote Target-Lesion Revascularization by Inducing Neoatherosclerosis After Stent Implantation. J. Am. Heart Assoc..

[12] Usui E., Yonetsu T., Ohmori M., Kanno Y., Nakao M., Niida T., Matsuda Y., Matsuda J., Umemoto T., Misawa T. (2022). Predictors of Near-Infrared Spectroscopy-Detected Lipid-Rich Plaques by Optical Coherence Tomography-Defined Morphological Features in Patients with Acute Coronary Syndrome. Front. Cardiovasc. Med..

[13] Ge J., Yu H., Li J. (2017). Acute Coronary Stent Thrombosis in Modern Era: Etiology, Treatment, and Prognosis. Cardiology.

[14] Chi G., AlKhalfan F., Lee J.J., Montazerin S.M., Fitzgerald C., Korjian S., Omar W., Barnathan E., Plotnikov A., Gibson C.M. (2024). Factors associated with early, late, and very late stent thrombosis among patients with acute coronary syndrome undergoing coronary stent placement: Analysis from the ATLAS ACS 2-TIMI 51 trial. Front. Cardiovasc. Med..

[15] Modi K., Soos M.P., Mahajan K. (2025). Stent Thrombosis. [Updated 2023 Jul 25]. StatPearls [Internet].

[16] Varenhorst C., Lindholm M., Sarno G., Olivecrona G., Jensen U., Nilsson J., Carlsson J., James S., Lagerqvist B. (2018). Stent thrombosis rates the first year and beyond with new- and old-generation drug-eluting stents compared to bare metal stents. Clin. Res. Cardiol..

[17] Torrado J., Buckley L., Durán A., Trujillo P., Toldo S., Valle Raleigh J., Abbate A., Biondi-Zoccai G., Guzmán L.A. (2018). Restenosis, Stent Thrombosis, and Bleeding Complications: Navigating Between Scylla and Charybdis. J. Am. Coll. Cardiol..

[18] Piccolo R., Efthimiou O., Varenne O., Baldo A., Urban P., Kaiser C., Remkes W., Räber L., de Belder A., van’t Hof A.W. (2019). Drug-eluting or bare-metal stents for percutaneous coronary intervention: A systematic review and individual patient data meta-analysis of randomised clinical trials. Lancet.

[19] Coronary Artery Stent Thrombosis: Clinical Presentation and Management—UpToDate. https://www.uptodate.com/contents/coronary-artery-stent-thrombosis-clinical-presentation-and-management/print.

[20] Pachl E., Zamanian A., Stieler M., Bahr C., Ahmidi N. (2021). Early-, Late-, and Very Late-Term Prediction of Target Lesion Failure in Coronary Artery Stent Patients: An International Multi-Site Study. Appl. Sci..

[21] Paramasivam G., Devasia T., Ubaid S., Shetty A., Nayak K., Pai U., Rao M.S. (2019). In-stent restenosis of drug-eluting stents: Clinical presentation and outcomes in a real-world scenario. Egypt. Heart J..

[22] Buchanan K.D., Torguson R., Rogers T., Xu L., Gai J., Ben-Dor I., Suddath W.O., Satler L.F., Waksman R. (2018). In-Stent Restenosis of Drug-Eluting Stents Compared with a Matched Group of Patients with De Novo Coronary Artery Stenosis. Am. J. Cardiol..

[23] Jakobsen L., Christiansen E.H., Freeman P., Kahlert J., Veien K., Maeng M., Ellert J., Kristensen S.D., Christensen M.K., Terkelsen C.J. (2025). Comparison of Outcome After Percutaneous Coronary Intervention for De Novo and In-Stent Restenosis Indications. Am. J. Cardiol..

[24] Naeem H., Khan U., Mohsin M., Niazi K., Malik J., Satti D.I., Anwar W. (2022). Comparison of Invasive Coronary Angiography Versus Computed Tomography Angiography to Assess Mehran Classification of In-Stent Restenosis in Bifurcation Percutaneous Coronary Intervention. Am. J. Cardiol..

[25] Elwany M.N., Abskharoun M., Dawood M., Al-Tahan S.M., Sanhoury M. (2024). The utility and effectiveness of the newer generation high-resolution coronary computed tomography angiography in the evaluation of coronary in-stent restenosis. Curr. Probl. Cardiol..

[26] Mitsis A., Eftychiou C., Kadoglou N.P.E., Theodoropoulos K.C., Karagiannidis E., Nasoufidou A., Ziakas A., Tzikas S., Kassimis G. (2024). Innovations in Intracoronary Imaging: Present Clinical Practices and Future Outlooks. J. Clin. Med..

[27] Yin D., Mintz G.S., Song L., Chen Z., Lee T., Kirtane A.J., Parikh M.A., Moses J.W., Fall K.N., Jeremias A. (2020). In-stent restenosis characteristics and repeat stenting underexpansion: Insights from optical coherence tomography. EuroIntervention.

[28] Yamamoto W., Fujii K., Otsuji S., Takiuchi S., Kakishita M., Ibuki M., Hasegawa K., Ishibuchi K., Tamaru H., Yasuda S. (2020). Optical coherence tomography characteristics of in-stent restenosis after drug-eluting stent implantation: A novel classification and its clinical significance. Heart Vessel..

[29] Erdogan E., Bajaj R., Lansky A., Mathur A., Baumbach A., Bourantas C.V. (2022). Intravascular Imaging for Guiding In-Stent Restenosis and Stent Thrombosis Therapy. J. Am. Heart Assoc..

[30] Serruys P.W., Cavalcante R., Collet C., Kappetein A.P., Sabik J.F., Banning A.P., Taggart D.P., Sabaté M., Pomar J., Boonstra P.W. (2018). Outcomes After Coronary Stenting or Bypass Surgery for Men and Women with Unprotected Left Main Disease. JACC Cardiovasc. Interv..

[31] Tocci G., Barbato E., Coluccia R., Modestino A., Pagliaro B., Mastromarino V., Giovannelli F., Berni A., Volpe M. (2016). Blood Pressure Levels at the Time of Percutaneous Coronary Revascularization and Risk of Coronary In-Stent Restenosis. Am. J. Hypertens..

[32] Zhang J., Zhang Q.M., Zhao K.M., Bian Y.-J.M., Liu Y.M., Xue Y.-T. (2022). Risk factors for in-stent restenosis after coronary stent implantation in patients with coronary artery disease: A retrospective observational study. Medicine.

[33] Huang X., Wang X., Zou Y., Chen S., Zhang R., Li L., Yu B., Hou J. (2017). Impact of Cigarette Smoking and Smoking Cessation on Stent Changes as Determined by Optical Coherence Tomography After Sirolimus Stent Implantation. Am. J. Cardiol..

[34] Liu X.H., Ma W.D., Zheng Y., Jia S., Fan Y.J., Yao Z.H., Zhang C.Y., Zhang Y., Hu Y.C., Ge M. (2018). Correlation between LDL-c to HDL-c cholesterol ratio and intracoronary in-stent restenosis. J. Xi’an Jiaotong Univ. Med. Sci..

[35] Wang J.-L., Qin Z., Wang Z.-J., Shi D.-M., Liu Y.-Y., Zhao Y.-X., Yang L.-X., Cheng W.-J., Zhou Y.-J. (2018). New predictors of in-stent restenosis in patients with diabetes mellitus undergoing percutaneous coronary intervention with drug-eluting stent. J. Geriatr. Cardiol..

[36] Azegami T., Kaneko H., Okada A., Suzuki Y., Fujiu K., Morita H., Takeda N., Fukui A., Yokoo T., Node K. (2024). Significance of eGFR and proteinuria for cardiovascular disease in individuals beyond 85 years of age. Nephrol. Dial. Transplant..

[37] Lee H.-F., Cheng Y.-W., Peng J.-R., Hsu C.-Y., Yang C.-H., Chan Y.-H., Chu P.-H. (2021). Impact of chronic kidney disease on long-term outcomes for coronary in-stent restenosis after drug-coated balloon angioplasty. J. Cardiol..

[38] Tariq S., Kumar R., Fatima M., Saghir T., Masood S., Karim M. (2019). Acute and sub-acute stent thrombosis: Frequency, predictors and features in patients undergoing primary percutaneous intervention at a tertiary care cardiac centre. Int. J. Cardiol. Heart Vasc..

[39] Li M., Hou J., Gu X., Weng R., Zhong Z., Liu S. (2022). Incidence and risk factors of in-stent restenosis after percutaneous coronary intervention in patients from southern China. Eur. J. Med. Res..

[40] Saleh A., Hammoudeh A., Tabbalat R., Al-Haddad I., Al-Mousa E., Jarrah M., Izraiq M., Nammas A., Janabi H., Hazaymeh L. (2016). Incidence and prognosis of stent thrombosis following percutaneous coronary intervention in Middle Eastern patients: The First Jordanian Percutaneous Coronary Intervention Registry (JoPCR1). Ann. Saudi Med..

[41] Kundu A., O’DAy K., Shaikh A.Y., Lessard D.M., Saczynski J.S., Yarzebski J., Darling C.E., Thabet R., Akhter M.W., Floyd K.C. (2016). Relation of Atrial Fibrillation in Acute Myocardial Infarction to In-Hospital Complications and Early Hospital Readmission. Am. J. Cardiol..

[42] Zimbakov Z., Zdravkovski I., Parisko E., Jovanova S. (2021). Riisk factors for in-stent restenosis in patients with percutaneous coronary interventions. J. Morphol. Sci..

[43] Wang P., Qiao H., Wang R., Hou R., Guo J. (2020). The characteristics and risk factors of in-stent restenosis in patients with percutaneous coronary intervention: What can we do. BMC Cardiovasc. Disord..

[44] Alexandrescu D., Crisan A., Mitu O., Macovei L., Costache I.I., Mitu I., Frasinariu O., Alexandrescu A., Georgescu C.A. (2021). Antiplatelet Therapy and Inflammatory Status Associated with Intra-Stent Restenosis after Percutaneous Coronary Intervention. Rev. Medico-Chir..

[45] Maehara A., Sugizaki Y. (2024). Intravascular Imaging for Guiding Percutaneous Coronary Intervention: What Does the Totality of Data Suggest, and Where Should We Go?. Circulation.

[46] Ali Z.A., Landmesser U., Maehara A., Shin D., Sakai K., Matsumura M., Shlofmitz R.A., Leistner D., Canova P., Alfonso F. (2024). OCT-Guided vs. Angiography-Guided Coronary Stent Implantation in Complex Lesions: An ILUMIEN IV Substudy. JACC.

[47] Liao J., Huang L., Qu M., Chen B., Wang G. (2022). Artificial Intelligence in Coronary CT Angiography: Current Status and Future Prospects. Front. Cardiovasc. Med..

[48] Fluder-Wlodarczyk J., Darakhovich M., Schneider Z., Roleder-Dylewska M., Dobrolińska M., Pawłowski T., Wojakowski W., Gasior P., Pociask E. (2025). Artificial Intelligence-Based Algorithm for Stent Coverage Assessments. J. Pers. Med..

[49] Dumitrașcu L.-M., Lespezeanu D.-A., Zugravu C.-A., Constantin C. (2024). Perceptions of the Impact of Artificial Intelligence among Internal Medicine Physicians as a Step in Social Responsibility Implementation: A Cross-Sectional Study. Healthcare.

